# Meaning and Attentional Guidance in Scenes: A Review of the Meaning Map Approach

**DOI:** 10.3390/vision3020019

**Published:** 2019-05-10

**Authors:** John M. Henderson, Taylor R. Hayes, Candace E. Peacock, Gwendolyn Rehrig

**Affiliations:** 1 Center for Mind and Brain, 267 Cousteau Place, University of California, Davis, CA 95618, USA; 2 Department of Psychology, University of California, Davis, CA 95618, USA

**Keywords:** attention, scene perception, eye movements

## Abstract

Perception of a complex visual scene requires that important regions be prioritized and attentionally selected for processing. What is the basis for this selection? Although much research has focused on image salience as an important factor guiding attention, relatively little work has focused on semantic salience. To address this imbalance, we have recently developed a new method for measuring, representing, and evaluating the role of meaning in scenes. In this method, the spatial distribution of semantic features in a scene is represented as a meaning map. Meaning maps are generated from crowd-sourced responses given by naïve subjects who rate the meaningfulness of a large number of scene patches drawn from each scene. Meaning maps are coded in the same format as traditional image saliency maps, and therefore both types of maps can be directly evaluated against each other and against maps of the spatial distribution of attention derived from viewers’ eye fixations. In this review we describe our work focusing on comparing the influences of meaning and image salience on attentional guidance in real-world scenes across a variety of viewing tasks that we have investigated, including memorization, aesthetic judgment, scene description, and saliency search and judgment. Overall, we have found that both meaning and salience predict the spatial distribution of attention in a scene, but that when the correlation between meaning and salience is statistically controlled, only meaning uniquely accounts for variance in attention.

## 1. Introduction

**Figure 1:**
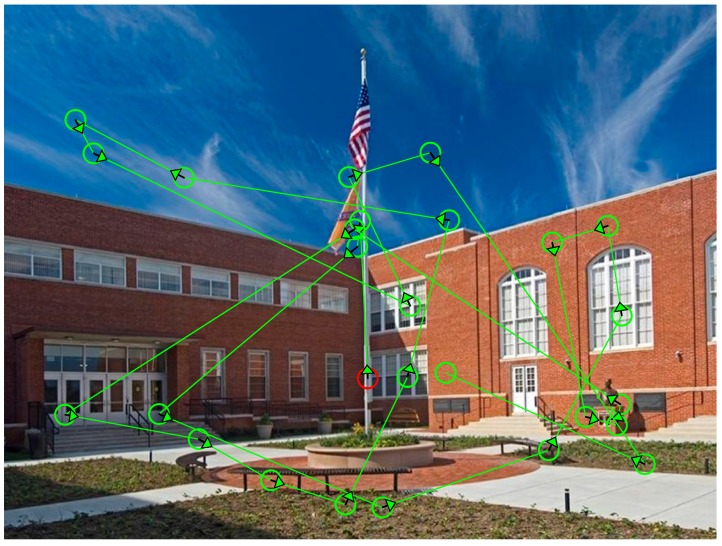
Scan pattern of a single viewer freely viewing a real-world scene.

The world contains an enormous amount of visual information, but human vision and visual cognition are severely limited in their processing capacity: Only a small fraction of the latent information can be analyzed at any given moment. Efficient visual cognition therefore requires selecting the information that is most relevant at the present moment for understanding and acting on the world. The primary way in which this selection takes place in natural vision is with overt attention via eye movements [1,2,3,4,5,6,7,8,9], as shown in Figure 1. Close or direct fixation of the scene region containing relevant information is typically necessary to perceive its visual details, to unambiguously determine its identity and meaning, and to encode it into short- and long-term memory. That is, what we see and understand about the world is determined by where we look [10].

Given the importance of eye movements for perception and cognition, a critical issue concerns understanding the representations and processes that guide the eyes through a visual scene in real time to support perception, cognition, and behavior [11]. Models based on image salience have provided an influential approach to eye movement guidance in scene perception. For static images, these models propose that attention is controlled by contrasts in primitive image features such as luminance, color, and edge orientation [12,13,14]. A key concept is the saliency map, which is generated by salience over the primitive features. Attention is then assumed to be captured or “pulled” to the most visually salient scene regions represented by the saliency map [15,16,17,18,19,20,21]. Because the primitive features are semantically uninterpreted, scene regions are prioritized for attentional selection based on image properties alone. The appeal of this type of image salience model is that visual salience is both neurobiologically plausible in the sense that the visual system is known to compute the assumed primitive features, and computationally tractable in the sense that working models have been implemented that generate image salience from these features [4].

We note that “salience” has different interpretations in different literatures, and so we want to be clear about which interpretation we are using. In vision science and attention research, the term is typically reserved for models in the Koch and Ullman tradition based on the idea that the human visual system computes difference maps from basic image features that are then combined and used to guide attention [19]. On the other hand, in computer vision and image processing, models that predict attention regardless of the underlying processes are also sometimes referred to as saliency models. This difference in usage can lead to confusion. For our purposes here, we specifically focus on image salience in the Koch and Ullman tradition, and to eliminate ambiguity, we use the terms “image salience” and “salience” to refer to that concept.

In contrast to models based on image salience, cognitive guidance models emphasize the important role of scene semantics in directing attention in scenes. In this view, attention is “pushed” by the cognitive system to scene regions that are semantically informative and cognitively relevant [10]. Cognitive guidance is consistent with evidence suggesting that viewers attend to semantically informative regions of a scene [5,6,22,23,24,25], as well as scene regions that are task-relevant [6,26,27,28,29,30,31,32,33,34]. For example, according to the cognitive relevance model [35,36], the attentional priority assigned to a scene region is based on its inherent meaning (e.g., “cup”) as well as its meaning with respect to the scene (e.g., “a cup in an office”) and the goals of the viewer (e.g., “I am looking for something I can put water into”). In the cognitive relevance model, the scene image is of course important: It is the input that enables these higher-level semantic processes and interpretations. Additionally, the scene image is the input for forming a map of potential attention targets. However, for this model, the critical hypothesis is that the image-based parse generates a “flat” (that is, unranked) landscape of potential targets rather than a landscape ranked by image salience. Attentional priority is then based on the detected and predicted informativeness (i.e., meaning) of the scene regions and objects in that landscape [3,4].

Until recently, it has been difficult to compare directly the influence of image salience and meaning on attentional guidance in scenes, because to do so requires representing both of them in a format that allows for comparable quantitative predictions of attentional distribution over the scene. Saliency map models naturally provide this type of prediction [17,18,20,21,30,37]. Unfortunately, it is far more difficult to create a computational model of meaning than it is to create a computational model of image salience, a likely reason that saliency models have been so popular [4,10]. Given this difficulty, studies of meaning-based models of attention in scenes have typically focused on manipulations of one or at most a small number of specific scene regions or objects [22,38,39,40,41]. However, these types of manipulations do not allow a direct comparison of image salience and semantic informativeness across the entire scene.

**Figure 2:**
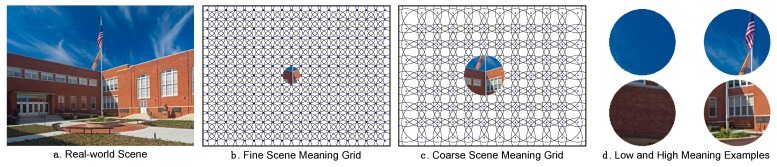
(**a**) A real-world scene; (**b**) fine scale, and (**c**) coarse scale patches from the patch grids; (**d**) examples of patches rated low (left column) and high (right column) in meaning.

**Figure 3:**
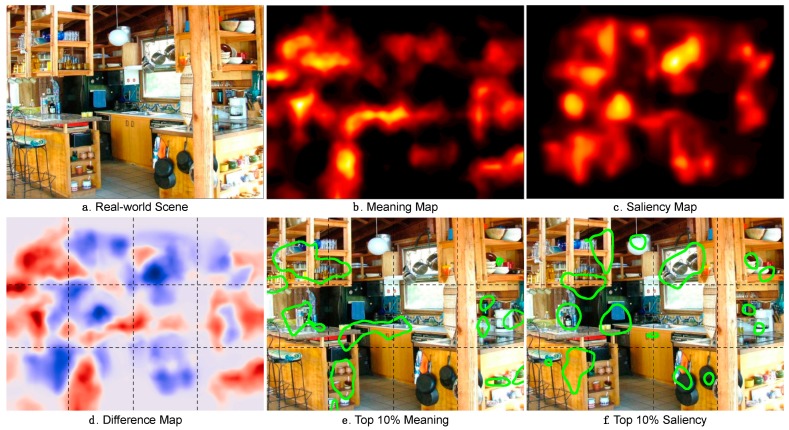
(**a**) Real-world scene; (**b**) scene’s meaning map; (**c**) saliency map; (**d**) difference map showing regions that are more meaningful (red) and salient (blue); (**e**) regions in the top 10% of meaning values; (**f**) regions in the top 10% of saliency values.

The issue we have recently pursued, then, is this: How can we generate and represent the spatial distribution of semantic informativeness over a scene in a format that supports direct comparison with a saliency map? To address this issue, we introduced a new approach based on meaning maps [42]. Meaning maps were inspired by two classic scene viewing studies [23,24]. The central idea of a meaning map is that it represents the spatial distribution of semantic informativeness over a scene in the same format as a saliency map represents the spatial distribution of image salience. To create meaning maps, we use crowd-sourced responses given by large numbers of naïve subjects who rate the meaningfulness of scene patches. Specifically, photographs of real environments are divided into dense arrays of objectively defined circular overlapping patches at two spatial scales (Figure 2). The two scales and numbers of patches are chosen based on simulations showing that we can recover ground truth visual properties of scenes from them [42]. Large numbers of workers on Mechanical Turk each rate a randomly selected subset of individually presented patches taken from the set of scenes to be rated. We then construct meaning maps for each scene by averaging these ratings by pixel over patches and raters and smoothing the results (Figure 3). Like image salience, meaning is spatially distributed non-uniformly across scenes, with some scene regions relatively rich in semantic informativeness and other regions relatively sparse. Meaning maps represent this spatial distribution pixel by pixel, and so offer a foundation for directly comparing the relative roles of meaning and image salience on attentional guidance.

In sum, meaning maps provide a conceptual analog of saliency maps by representing the spatial distribution of semantic features associated with informativeness across a scene. Meaning maps generate predictions concerning attentional guidance that can be tested using the same methods that have been used to test predictions from saliency maps. This allows contrasting predictions from semantic informativeness and image salience to be directly compared [42].

## 2. Review of Results

Meaning maps and saliency maps serve as predictions of how attention will be guided through scenes. The key empirical question is how well these predictions accord with observed distributions of attention produced by people viewing scenes. 

**Figure 4:**
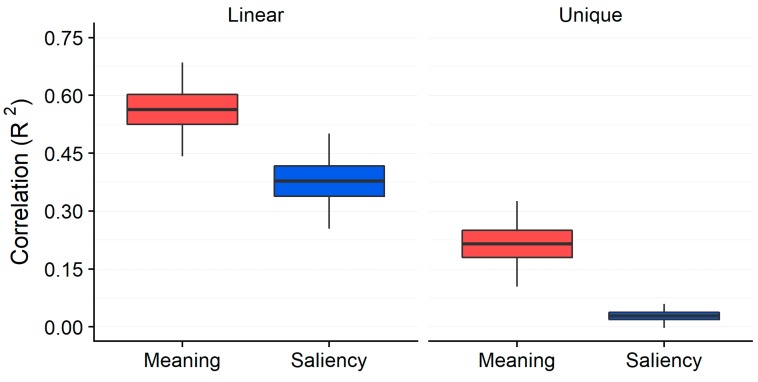
Squared linear correlation and semi-partial (unique) correlation for meaning and image salience across 40 scenes from a scene memorization task in Henderson and Hayes (2017) [42]. Box plots show the grand mean (black horizontal line), 95% confidence intervals (colored box), and one standard deviation (black vertical line).

In a first study directed toward this question, we asked subjects to view a set of scenes for 12 s each while their eye movements were recorded [42]. Subjects viewed the scenes to prepare for memory and aesthetic judgment questions that were presented after viewing. We operationalized attention maps as fixation density maps that reflected the spatial distribution of eye fixations across the scene. Importantly, the attention maps represented attention in the same format and on the same scale as the meaning and saliency maps. We then contrasted the degree to which the spatial distribution of meaning and salience predicted viewers’ attention over the scenes. The results showed that both meaning and salience predicted attention, but that when the association between meaning and salience was statistically controlled with semi-partial correlations, only meaning uniquely accounted for attentional variance. Furthermore, the influence of meaning was observed both at the very beginning of scene viewing and throughout the trial. Figure 4 summarizes the data from this study. This result was observed in both scene memorization and aesthetic judgment viewing tasks. Given the strong observed correlation between meaning and salience, and the finding that only meaning accounted for variance in attention when that correlation was controlled, we concluded that the existing data are consistent with a theory in which meaning is the main factor guiding attention through scenes.

In our initial study, we focused on the spatial distribution of attention by measuring the locations of fixations [42]. However, fixations also differ by duration, and fixation duration is known to reflect ongoing visual and cognitive activity [43,44,45,46,47,48,49]. Therefore, in a reanalysis of the data from Henderson and Hayes (2017), we examined the relative influences of semantic features and image salience on the distribution of attention taking into account attentional dwell time at each location [42,50]. This was accomplished by creating attention maps that weighted each fixation by its duration. Again, the question was whether these duration-weighted attention maps would be best accounted for by meaning maps or saliency maps. Using the same analysis methods with these duration-weighted attention maps, we replicated all of the basic data patterns that we observed in the original study. Specifically, we found that both meaning and salience were associated with attention, but that when the correlation between meaning and salience was statistically controlled, only meaning accounted for variance in attention. Once again, the influence of meaning was observed both at the beginning of scene viewing and throughout the trial. Therefore, whether the measure of attention is based on location only or includes dwell time, the answer with respect to meaning and image salience is the same [50].

In our initial experiments demonstrating the advantage of meaning maps over saliency maps, subjects viewed scenes in order to prepare for memory and aesthetic judgment questions that were presented after viewing [42,50]. In those tasks, the responses were off-line with respect to scene perception, so viewers may not have guided attention as quickly or under as much control as they might in a task requiring real-time responses during viewing. Therefore, in the next study we investigated how well meaning and image salience account for attention when the subject is actively engaged with and responding to the scene continuously in real time, and the guidance of attention is directly relevant to the real-time task. For this purpose, we used two scene description tasks [51].

We drew on evidence that language production is incremental in the sense that speakers interweave planning and speaking instead of planning the entire production and then executing the plan [52]. That is, speakers typically plan and produce small units of speech (words and phrases) that are tied to each scene region that they fixate. Scene description therefore allows us to examine the relative influences of semantic information and image salience under conditions in which on-line changes in attention to specific scene regions are functional and necessary. We used this basic paradigm in two experiments. In one experiment, subjects described what someone might do in each scene, and in a second experiment, they simply described each scene. In both experiments, subjects were asked to begin their description when the scene appeared and to continue speaking for 30 s of scene presentation. Their eyes were tracked throughout each trial. The main result was that, once again, both meaning and salience were associated with the spatial distribution of attention, but when the correlation between meaning and salience was statistically controlled, only meaning accounted for variance in attention. This basic result was seen in both experiments. Therefore, once again we found no evidence for a unique influence of image salience on attention that could not be explained by the relationship between image salience and meaning, whereas the unique influence of meaning could not be attributed to salience.

So far, across all four tasks we have described (memorization, aesthetic judgment, scene description, and action description), meaning was highly relevant. Perhaps for tasks in which image salience is necessary and meaning is irrelevant, salience would better predict attention. To test this hypothesis, in a final set of experiments we examined the role of meaning and salience in two experiments using tasks for which meaning was completely irrelevant and saliency was critical: a brightness rating task in which participants rated each scene for its overall brightness, and a brightness search task in which participants counted the number of bright patches in each scene [53]. If meaning was used to guide attention in the previous tasks because those tasks emphasized the semantic content of the scenes, then the relationship between meaning and attention should no longer hold in the tasks that focus on the image itself. On the other hand, if the use of meaning to guide attention is a fundamental property of the operation of the attention system when faced with real-world scenes, then we should continue to see a relationship between meaning and attention even if meaning is irrelevant. Using the same methods as the previous studies, the striking finding was that in both the brightness rating and brightness search tasks, the results were very similar to the prior experiments: When the correlation between meaning and salience was controlled, only meaning uniquely accounted for significant variance in attention. These results showed that the relationship between meaning and attention is not restricted to viewing tasks that require the viewer to analyze meaning. The results support theories in which scene semantics play a central role in attentional guidance in scenes.

## 3. Discussion

We have reviewed the meaning map approach and shown how we have used it to investigate the relative roles of semantic informativeness and image salience on attentional guidance. Our results strongly suggest a fundamental and mandatory role for meaning in attentional guidance in real-world scenes. These results are consistent with a growing understanding that both overt and covert visual attention are often under the influence of the meaning of the visual stimulus, even when that meaning seems irrelevant to the task. Examples of this type of semantic effect are influences of object meaning on eye movements in the visual world paradigm [54], and influences of semantic object relationships on covert attention [55]. Furthermore, we found that the observed relationship between meaning and attention was about as strong as it could be given the variability in attention maps across subjects [42]. In the remainder of this section we consider and discuss some additional related issues.

First, although it has sometimes been proposed that image salience and semantic content are likely to be correlated in scenes, it has been difficult to test this hypothesis directly. The meaning map approach provides such a method. Further, as we have shown across several studies, that correlation is robust. An important implication of this finding is that previous results demonstrating a relationship between saliency maps and attention cannot be taken as evidence for a functional role of salience in guiding attention. Indeed, as we have reviewed above, essentially the entire relationship between image salience and attention can be attributed to the association between image salience and semantic content.

Second, it is important to be clear that meaning maps are not a theory of scene semantics. They are simply a method for tapping into and representing human judgments concerning the relative informativeness of scene regions continuously over space. Meaning maps provide an operational definition of the spatial distribution of meaning that can be quantified, but they do not offer direct insight into the nature of scene semantics or how meaning is represented in the mind and brain. 

That said, it may be that the meaning map approach can be used as a tool for beginning to get a handle on the nature of scene semantics. For example, the type of scene meaning we have investigated so far has been based on ratings of scene patches that were presented to raters independently of the scenes from which the patches were taken. These experiments therefore focus on the role of what we call scene-intrinsic context-free meaning on attention [51]. We might collect ratings using other types of questions that could provide insight into other types of semantic representations. For example, we could compare context-free to contextualized meaning in which the degree of meaning associated with a scene region is based on its relationship to the scene as a whole rather than on its own. Similarly, we might consider goal-related meaning, in which the meaning of a scene region is based on its relationship to the viewer’s goals rather than intrinsic to the scene itself. Using judiciously chosen ratings, we might be able to unravel how different types of meaning are related to each other and to performance over different perceptual and cognitive tasks. The meaning map approach provides a method for pursuing these questions. Relatedly, the meaning maps we have investigated so far based on context-free ratings may not be the type of meaning most associated with attention, and we may therefore be underestimating the relationship between semantic features and attention.

One advantage of saliency maps over meaning maps is that the former are image computable: They can be derived automatically and without human intervention from computational models. In comparison, meaning maps are not image computable and require human raters. For this reason, one might suggest that saliency models are to be preferred as an explanation of human attention. From an engineering perspective, this view has merit. However, from a vision and cognitive science perspective, it does not. In our view, the interesting psychological claim of the image salience hypothesis is that human attention in real-world scenes is driven by contrasts in basic image features. This claim has been supported by a large number of experiments showing a correlation between saliency maps and human fixations. The alternative hypothesis we are pursuing is that image salience effects are actually disguised meaning effects, because image features and semantic features in scenes are correlated [42]. Indeed, this is what we find, with very little if any unique variance accounted for by salience once the variance accounted for by semantic features is controlled. This comparison is not one of a computational model versus human ratings, but one of two competing psychological theories. That is, we are concerned with psychological principles here, not modeling. There is no logical requirement that testing this (or any) psychological hypothesis requires image computable semantic features. Furthermore, until we have a computational model of the entire semantic system (which is clearly a long way off), there is no other way to go about comparing salience to semantics. Saliency models have been influential because they have been the only game in town [4,10]. Meaning maps provide an alternative game, but their creation does require human judgment. From our perspective, this approach is similar to the approach that uses human labeling to parse and label objects in scenes as in the labelMe database [56] to produce object ground truth. Of course, it would be very interesting to use the ground truth represented by meaning maps to try to train a model to find meaningful regions (and indeed we are pursuing this idea), but that is not necessary for testing the theories at stake.

Relatedly, it has been argued that Koch and Ullman-inspired saliency models like GBVS are no longer state of the art, but instead have been replaced by a newer class of models based on deep neural networks (DNNs) that have recently been found to predict human attention quite well [57]. Given this, one might ask why we should take standard saliency models as the baseline for comparison to meaning. In our view, although these DNN models are impressive from the perspective of pushing the boundaries of deep learning and big data, it is not clear at this point how much they have to say about active biological vision. For example, DNN models are trained on fixations over one set of scenes and then predict fixations on another set of scenes. It is clear that humans do not learn where to fixate based on supervised learning from the fixations of others or by ingesting large amounts of external fixation data. It is also unclear whether the mechanisms used by DNNs to predict attention operate on the same principles that are used by the human brain. For these reasons, although we watch developments in this field with great interest, we are not yet sure how their successes and failures should be interpreted from the perspective of human attentional processes.
